# Mental health prevention: moving forward with the evidence

**DOI:** 10.1007/s44192-025-00346-8

**Published:** 2025-11-27

**Authors:** Marco Colizzi

**Affiliations:** 1 Unit of Psychiatry and Eating Disorders, Department of Medicine (DMED), University of Udine, 33100 Udine, Italy; 2 Department of Psychosis Studies, Institute of Psychiatry, Psychology and Neuroscience, King’s College London, London, SE5 8AF UK

The global burden of mental health challenges continues to escalate. This trend is intensified by societal transformations, technological advances, pandemics, and shifting economic landscapes [1]. As we strive to move from reactive to proactive approaches in mental health, it is imperative to root our efforts in solid evidence [2]. This topical collection, *Mental Health Prevention: Moving Forward with the Evidence*, brings together a diverse and timely collection of studies and reviews that illuminate the complexities, challenges, and innovations in mental health prevention today (https://link.springer.com/collections/feadjbdgje).

The intersection between technology and mental well-being is an emerging frontier [3]. Research shows that human factors often overlooked in cybersecurity management can contribute to psychological strain and reduced productivity, even when digital systems are well-designed. Interventions such as digital detox strategies can help alleviate these burdens, offering practical pathways to promote mental health in the workplace.

The long tail of the COVID-19 pandemic continues to affect mental health in profound ways [4]. Large-scale studies highlight a strong and enduring link between prolonged infection and heightened risks for anxiety and depression. These findings provide crucial guidance for policymakers and healthcare providers. Complementing this, prospective research tracing psychological symptoms from pregnancy to postpartum during the pandemic underscores persistent vulnerabilities among perinatal populations. Additional studies show that enhancing mental health literacy in postpartum populations can offer preventive benefits. Together, these findings emphasize the importance of sustained monitoring and tailored support for at-risk groups.

Psychological complexities also extend to major medical events beyond infectious diseases [5]. Evidence indicates that psychosocial evaluations and sustained emotional support are central to successful outcomes in organ transplantation. Similarly, screening for anxiety, depression, and personality traits in patients with chronic conditions such as ischemic heart disease ensures a more holistic approach to care.

Youth mental health prevention remains a critical pillar for the future [2]. Studies show that young people employ diverse strategies to cope with emotional distress, revealing community- and policy-level opportunities to bolster resilience. Research on mental health literacy among school-attending youth highlights both progress and persistent gaps, pointing to evidence-based avenues for fostering knowledge and coping skills early in life.

Educational environments also play a pivotal role [6]. Findings from health science students indicate that academic pressures contribute significantly to stress. Systemic reforms, such as mentoring programs, workload adjustments, and structured peer support, are needed to safeguard the well-being of future healthcare professionals and build a resilient workforce.

Innovative treatments for persistent public health challenges are gaining attention [7]. Systematic reviews on psychedelics as potential interventions for tobacco use disorder demonstrate how revisiting ancient compounds with modern research methods can open new therapeutic possibilities when traditional approaches fall short.

Addressing structural determinants of mental health is equally essential [8]. Research on the psychological impact of war reveals how conflict and displacement exacerbate mental health challenges among children and adolescents. Real-world programs, such as trauma-informed and culturally adapted school-based interventions in refugee communities, illustrate how evidence can be translated into effective preventive practice. Broader studies advocate for coordinated, system-level efforts, such as integrating mental health services into primary care, expanding community-based support, and leveraging digital tools for early detection. These efforts should be accompanied by ethical safeguards and inclusivity. Forward-looking commentaries identify priorities for research, policy, and practice, emphasizing evidence-based interventions, structural reform, and sustainable collaboration.

Taken together, the articles in this topical collection illustrate that mental health prevention is a multidimensional effort requiring collaboration across disciplines, sectors, and societies. Moving forward, prevention strategies must be grounded not only in robust evidence but also in compassion, cultural sensitivity, and structural innovation.

We hope this collection inspires researchers, practitioners, policymakers, and community leaders to continue advancing the frontiers of mental health prevention—ensuring that the future of mental health is not only about treating illness but about fostering well-being from the start.
